# Rapid Bead-Based Antimicrobial Susceptibility Testing by Optical Diffusometry

**DOI:** 10.1371/journal.pone.0148864

**Published:** 2016-02-10

**Authors:** Chih-Yao Chung, Jhih-Cheng Wang, Han-Sheng Chuang

**Affiliations:** 1 Department of Biomedical Engineering, National Cheng Kung University, Tainan, Taiwan; 2 Division of Urology, Department of Surgery, Chi Mei Medical Center, Tainan, Taiwan; 3 Medical Device Innovation Center, National Cheng Kung University, Tainan, Taiwan; Tsinghua University, CHINA

## Abstract

This study combined optical diffusometry and bead-based immunoassays to develop a novel technique for quantifying the growth of specific microorganisms and achieving rapid AST. Diffusivity rises when live bacteria attach to particles, resulting in additional energy from motile microorganisms. However, when UV-sterilized (dead) bacteria attach to particles, diffusivity declines. The experimental data are consistent with the theoretical model predicted according to the equivalent volume diameter. Using this diffusometric platform, the susceptibility of *Pseudomonas aeruginosa* to the antibiotic gentamicin was tested. The result suggests that the proliferation of bacteria is effectively controlled by gentamicin. This study demonstrated a sensitive (one bacterium on single particles) and time-saving (within 2 h) platform with a small sample volume (~0.5 μL) and a low initial bacteria count (50 CFU per droplet ~ 10^5^ CFU/mL) for quantifying the growth of microorganisms depending on Brownian motion. The technique can be applied further to other bacterial strains and increase the success of treatments against infectious diseases in the near future.

## Introduction

Sepsis is a fatal disease that claims thousands of lives every year and ranks in the top 10 leading causes of death worldwide [1]. Antimicrobial susceptibility testing (AST) plays a pivotal role in the success of sepsis treatments. The gold standard of AST, broth microdilution, relies on the growth of microorganisms and is commonly measured according to turbidity limited to at least 10^7^ CFU/mL [2]. However, the turnaround time for conventional AST generally takes more than 24 h [3, 4], resulting in high patient mortality [5]. Moreover, the empirical antibiotic therapies administered before pathogen identification and the long wait for the AST outcome are likely to cause high failure rates and the spread of multi-resistant pathogens [2, 6]. Molecular diagnostic techniques used for detecting resistant genes, such as multi-PCR, are time-saving [4] yet unsuitable for determining the minimum inhibitory concentration (MIC) and unknown antibiotic resistance genes [2]. Consequently, timely, effective, and efficient screening of drug susceptibility is crucial to saving lives and controlling the proliferation of superbugs [2, 6, 7].

For enhancing the efficiency of AST, techniques based on morphological analysis, [8] fluorescence intensity [9], dielectrophoresis [10], Raman-enhanced spectra [11, 12], atomic force microscopy [13], asynchronous magnetic bead rotation (AMBR) [14, 15], and microfluidic devices [3, 16–18] have been reported in recent years. Particularly, the AMBR sensor was a very rapid tool for determining the MIC of gentamicin on *Escherichia coli* [14, 15]. In measuring four doses of gentamicin, previous studies [14, 15] about the AMBR sensor have significantly reduced the AST measurement time from more than 1 day to 15–30 min. However, a sophisticated driving magnetic field was still required for determining the spinning period of each magnetic bead. Among these techniques, 0.5–4 h are typically required for a complete AST process. Nevertheless, a limited scope of applications, inconsistency with conventional methods, complex fabrication procedures, and requirements of high-end equipment remain the major concerns regarding their clinical use. Recently, bead-based immunoassays have emerged because of great flexibility, a large reaction surface area, a small sample quantity, and simultaneous determination of multiple pathogens [19, 20]. Considering these advantages, combining optical diffusometry with immunoassays has been effective for monitoring the growth of bacteria. Gorti et al. first carried out a proof of concept in a pilot study on detecting M13 viruses [21]. They explored the possibility of detecting and quantifying pathogens by measuring the change of particle diffusivity. Their result indicated that the particle diffusivity decreased when the particle size increased because of the target analytes. Similarly, the feasibility of detecting C-reactive protein (CRP), a risk factor for cardiovascular diseases, by using microparticle tracking velocimetry in various viscosity solutions was investigated by Fan et al [19, 20]. They reported detecting CRP concentrations as low as 0.1 μg/mL. These successful attempts have proven the potential of applying Brownian motion to quantify low-abundance microorganisms.

In this study, a technique combining optical diffusometry and bead-based immunoassays was developed for quantifying the growth of specific microorganisms and achieving rapid AST. Optical diffusometry requires only a microscope and a camera to quantify the Brownian motion of particles. Because Brownian motion is a random and self-driven physical phenomenon, this technique can avoid the aforementioned limitations [22]. In our concept of design, as bacteria grow and attach to particles, the measured Brownian motion tends to vary in response to the increased equivalent particle diameter. When bacteria are sensitive to an antibiotic, the change will then be halted, which can be associated with the minimum inhibitory concentration (MIC) of the drug. In an attempt with *P*. *aeruginosa*, we demonstrated that an AST process can be complete within 2 h. In addition, the minimum requirement of the sample volume is only 0.5 μL while the initial bacteria count is as low as 50 CFU per droplet (10^5^ CFU/mL). We believe more applications relevant to quantification of other microorganisms, such as water quality monitoring, can also be implemented in the same fashion.

## Materials and Methods

### Reagents and Bacteria

*Pseudomonas (P*.*) aeruginosa* (ATCC 27853), a Gram-negative motile bacterium strain (0.5–1 × 1.5–3 μm), was a gift from Dr. H.C. Chang of Department of Biomedical Engineering at Nation Cheng Kung University, Taiwan. Carboxylate-/amine-modified polystyrene particles (*d*_p_ = 2 μm, 1.05 g/cm^3^, Ex: 520 nm/Em: 540 nm), 1-ethyl-3-(3-dimethylaminopropyl) carbodiimide (EDC), N-hydroxysuccinimide (NHS), 2-(n-morpholino)-ethanesulfonic acid (MES), and gentamicin were obtained from Sigma-Aldrich (St. Louis, MO, USA). The particles were used as probes to detect the concentration of bacteria in a sample suspension. Gentamicin was employed as an antibiotic in the AST assessment. Anti-*P*. *aeruginosa* polyclonal antibody (ab67905) and Anti-*Staphylococcus aureus* polyclonal antibody (ab20920) were ordered from Abcam (Cambridge, UK). Tryptic soy broth (TSB) came from BD (East Rutherford, NJ, USA). *P*. *aeruginosa* bacteria were incubated in TSB at 37°C for 12–16 h before use. Dead *P*. *aeruginosa* bacteria were obtained by exposing the bacteria under 8W 254 nm UV light (EBF-280C, Spectroline^®^) for 24 h. When conducting AST, gentamicin was mixed with a drop of bacteria to achieve a range of final concentrations from 0.02 to 0.2 μg/mL.

### Optical Diffusometry

**Fig 1:**
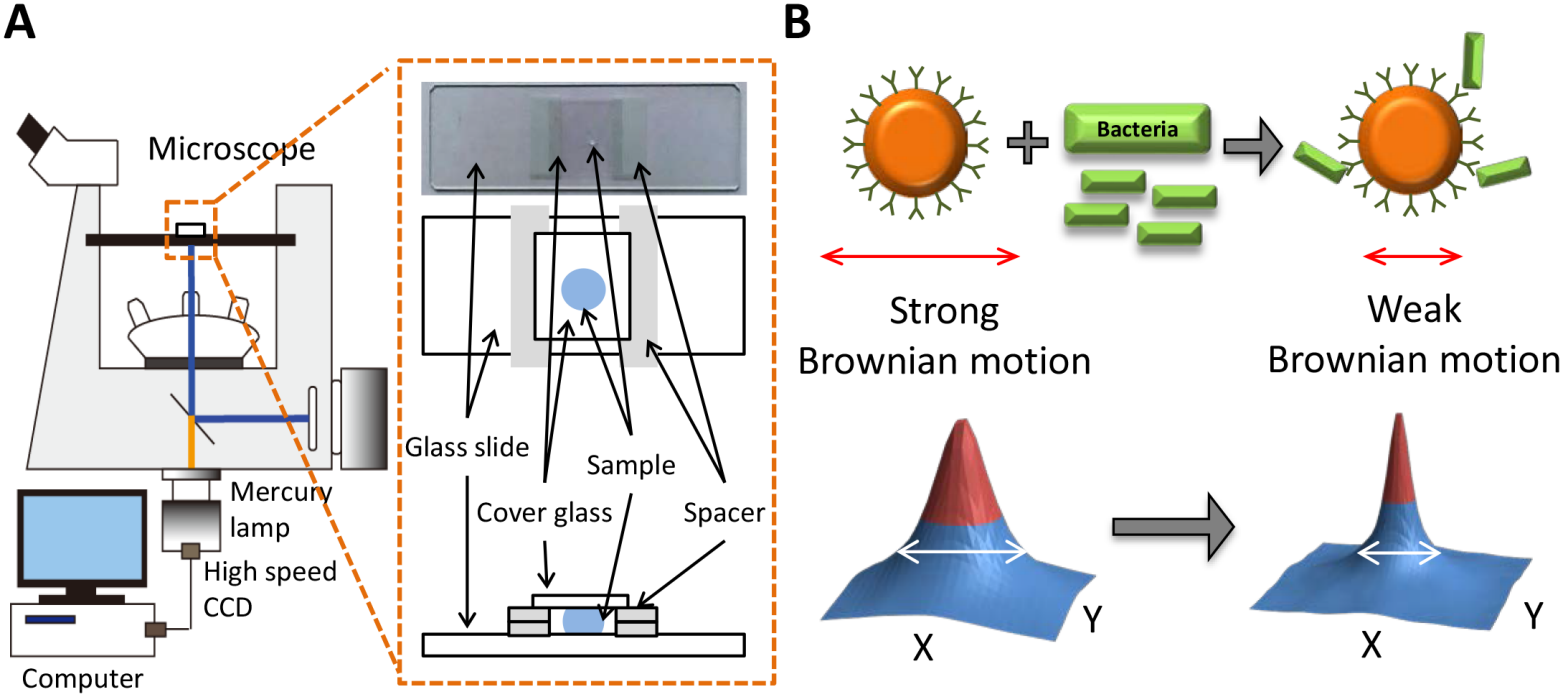
The optical diffusometric platform. (A) Schematic of the optical diffusometry. (B) The relationship of Brownian motion and the particle size change due to the bacterium-particle binding. The corresponding diffusivity values are derived from the cross-correlation algorithm. A large particle diameter results in a narrow correlation peak.

In this study, a fluorescent microscope (IX71, Olympus) and a high-speed camera (GX3, NAC Image Technology) were integrated to build an optical diffusometric platform (Fig 1A). A sample suspension containing modified particles and bacteria were loaded on a glass slide covered by a cover glass with a spacer of 110 μm [22]. The image plane was focused in the middle of the gap to avoid hindered diffusion. A series of particle images were recorded with a 20× or 40× objective at a frame rate of 10 Hz for 20 s. For each AST measurement, the recording was performed every 20 min in a total timespan of 2 h.

Brownian motion is random movement of particles subject to ambient temperature, liquid viscosity, and particle size. The random displacement, *x*, of a particle caused by Brownian motion is associated with the time interval, Δ*t*, and the diffusion coefficient, *D*. The relation has been described by Langevin [23] and Einstein [24] as

$$\langle x^{2}\rangle =2D\Delta t,$$ (1)

where

$$D=\frac{k_{B}T}{3\pi \mu d_{p}},$$ (2)

*k*_*B*_ is the Boltzmann constant, *T* is the absolute temperature of the fluid, *μ* is the viscosity of the fluid, and *d*_p_ is the particle diameter. At constant temperature and liquid viscosity, the diffusion coefficient is simply a function of the particle diameter. Accordingly, as bacteria grow and attach to particles, the measured Brownian motion will change with the particle size (Fig 1B).

Although polystyrene particles used in this study have a density close to water (*ρ* = 1.05 g/cm^3^), particle sedimentation may still disturb the diffusivity when particles are near the bottom wall. Here the Stokes sedimentation velocity (*ν*_*S*_ = 2Δ*ρgR*^*2*^*/9μ*) is estimated to be 1.15 × 10^−1^ μm/s when particles with a radius of 1 μm are suspended in a water solution (viscosity *μ*≃1 cP). Considering the particle images were recorded for only 20 s, the sedimentation distance (2.3 μm) during the measurement was then negligible [25]. In addition, the horizontal hindered diffusion coefficient is calculated to evaluate the interferences from the bottom and top walls [22]:

$$\beta_{∥}\equiv \;\frac{D_{∥}}{D}\approx \left[1-\frac{9}{16}\left(\frac{d_{\text{p}}}{2h}\right)+\frac{1}{8}{\left(\frac{d_{\text{p}}}{2h}\right)}^{3}\right],$$ (3)

where *h* is the distance between the particle and the wall; *D* and *D*_*║*_ represent the bulk diffusion and the component of the hindered diffusion parallel to the wall, respectively. In Eq (3), the high-order terms are neglected. Considering that *d*_p_ = 2 μm and *h* = 55 μm (the measurement plane is focused in the middle of the chamber) in the study, the hindered diffusion coefficient, *β*_*║*_, is estimated to be as high as 0.99. As a result, the hindered diffusion herein is negligible.

### Cross-Correlation Algorithm

**Fig 2:**
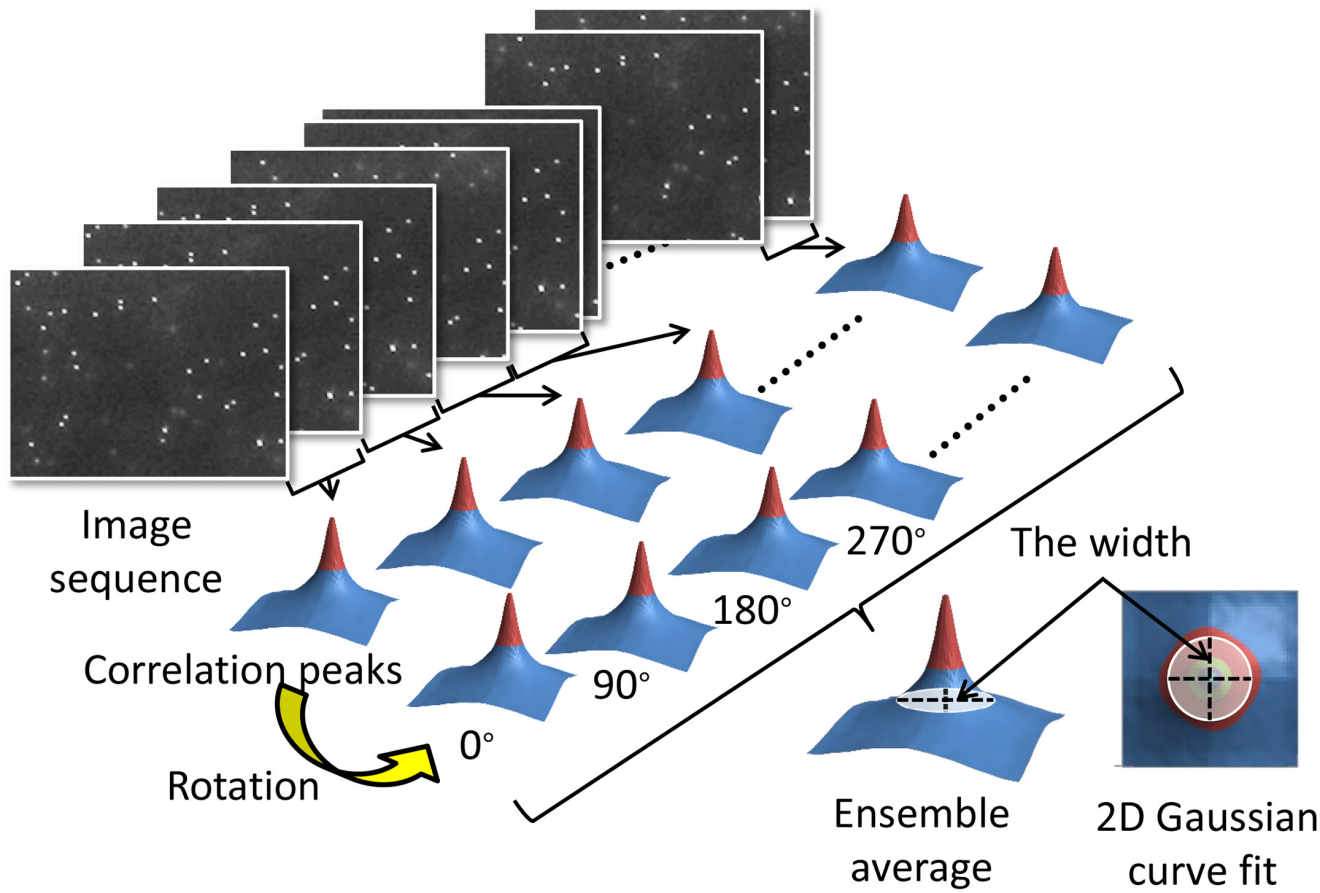
Conceptual diagram of the computational procedure of the cross-correlation algorithm for a series of particle images. The particle images were analyzed by spatially cross-correlation algorithm and summed up in the correlation domain to achieve an ensemble average.

A spatial cross-correlation algorithm was used here to calculate the degree of Brownian motion of consecutive particle images in Matlab (Fig 2) [22, 26]. To achieve a high correlation, an image pair of the flow field acquired at times *t*_*1*_ and *t*_*2*_ = *t*_*1*_ + *Δt* are calculated each time. By denoting the first image as *I*_1_(*X*) and the second image as *I*_2_(*X*), the cross-correlation function can be obtained using the convolution integral [26]:

$$R\left(S\right)=\int I_{1}\left(X\right)I_{2}\left(X+s\right)dX.$$ (4)

*R*(*S*) can be decomposed into three components as

$$R\left(S\right)=R_{C}\left(s\right)+R_{F}\left(s\right)+R_{D}\left(s\right),$$ (5)

where *R*_*C*_(*s*) is the convolution of the intensities of the two images and is a function of *s* with its diameter equal to the particle diameter, *R*_*F*_(*s*) is the fluctuating noise component, and *R*_*D*_(*s*) is the displacement component of the correlation function and gives the distance traveled by the particle during time Δ*t*. Hence *R*_*D*_(*s*) is the component of the correlation function which contains the velocity information.

The shape and height of the correlation function in the presence of Brownian motion assume a Gaussian shape and its center locates the mean particle displacement. [22, 26] The displacement and the width of the correlation function depend on the probability function *f* (*x*′,*t*_2_;*x*,*t*_1_) where *f* denotes the probability for a particle initially at *(x*,*t*_*1*_*)* to move into the volume (*x′*, *x′ + dx*) at *t*_*2*_. [26] This change in *f* due to Brownian motion has the effect of broadening the correlation function and reducing its height. In our method, the increase in the width of the correlation function contains the information that yields the equivalent volume diameter of a particle attached to bacteria. [22, 26]

In practice, a Fast Fourier Transform is applied to calculate the image pairs for a correlation peak. Subsequently, the width of the correlation peak is defined by 1/*e* intensity of the Gaussian distribution (*i*.*e*., *e* is the base of natural logarithm) which is derived from fitting the intensity profile with a two dimensional Gaussian curve. The widths of the correlation peaks, Δ*S*_*a*_^*2*^ and Δ*S*_*c*_^*2*^, are derived from the auto-correlation and the cross-correlation functions, respectively. The relation between the cross-correlation functions and the diffusion coefficient (Eq 2) can then be expressed as

$$D\propto \frac{\Delta {\text{S}}_{c}^{2}-\Delta {\text{S}}_{a}^{2}}{△t},$$ (6)

Theoretically, a broader correlation peak implies stronger diffusivity. Hence, the equivalent particle diameter can be determined by identifying the width of a correlation peak from paired particle images. Diffusivity is introduced as a statistical measure used to quantify Brownian motion. The diffusivity here is defined as a ratio of the squared difference between the peak widths of the cross-correlation and the autocorrelation $\left(\Delta S_{c}^{2}-\Delta S_{a}^{2}\right)$ to the time elapsed (Δ*t*).

An ideal correlation peak should locate in the center of the correlation domain due to the stationary colloidal suspension. Considering the motility of *P*. *aeruginosa*, however, a correction step to offset the correlation bias resulting from the microorganism’s locomotion is required. To this end, we sequentially rotated each correlation domain by 90° for a series of consecutive correlation peaks to achieve an ensemble average. At last, the result showed that the bias and uncertainty were effectively reduced.

### Bead-based Immunoassay

Through antigen-antibody reactions, bead-based immunoassays were employed to detect specific bacteria in physiological fluids. The carboxyl groups on the anti-*P*. *aeruginosa* polyclonal antibody were activated by incubation with 10 mg/mL EDC and 10 mg/mL NHS at a mole ratio of 1:400:1200 for 15 min. Amine-modified polystyrene particles were used as sensing probes for *P*. *aeruginosa*. The particles were initially washed with a MES buffer (pH 5.5) to prevent agglomeration. The polystyrene particles were then functionalized with the EDC-NHS activated antibodies at 4°C and 800 rpm for 4 h to achieve a final volumetric concentration of 0.625% v/v (S1 Fig). To assess the binding efficacy between the particles and the bacteria, the antibody-conjugated particles (0.025%, 5.7 × 10^7^ beads/mL) were incubated with *P*. *aeruginosa* (10^9^ CFU/mL) for 1 h and then measured under an optical microscope for another 1 h. Scanning electron microscopy (SEM, JEM6700, JEOL) was used to confirm the bacterium-particle complex. In addition, for understanding the nonspecific binding, non-modified, carboxylate-modified, amine-modified, and anti-*S*. *aureus* polyclonal antibody modified particles were separately incubated with *P*. *aeruginosa* (10^9^ CFU/mL) and then measured their binding rates under an optical microscope.

### Quantification of Bacteria by Diffusometry

To verify the theoretical relationship between the diffusivity and the bacterium-particles complex, a small liquid droplet (~0.5 μL) containing particles attached to live or dead bacteria was prepared for each measurement. The dead bacteria were obtained by sterilizing live bacteria with 8 W 254 nm UV light at 4°C for 24 h. Image processing was performed after the bacterium-particle complex was complete. For analysis of single particles attached to various numbers of bacteria, particle images (image sets n = 10 in each group) were recorded every 0.1 s with a 40× objective after 1 h of incubation with *P*. *aeruginosa*. By contrast, images of a population of particles incubated with *P*. *aeruginosa* at ratios of 1:10, 1:1, 1:0.1, 1:0.01, and 1:0 were recorded every 0.1 s with a 20× objective (image sets n = 5 in each group). A mean diffusivity value of each group was obtained through the cross-correlation algorithm. Because variations of measurements may result from different environmental conditions or background noise, relative diffusivity values are more suitable than absolute ones. All measurements were at last divided by the diffusivity of free particles (S_p_) to get relative values (S/S_p_).

### Equivalent Volume Diameter

For determining the interactions between antibody-conjugated particles and dead bacteria, a theoretical model assuming that the diffusivity changes resulting from the bacterium-particle binding effect was employed. In the model, the bacterium-particle complex forms a composite structure whose diameter can be defined according to the equivalent volume diameter [27]:

$$d_{v}={\left(\frac{6V}{\pi }\right)}^{1/3},$$ (7)

where *V* is the total volume of the attached bacteria and the carrier particle.

### Assessment of Rapid Antimicrobial Susceptibility Testing

An AST process was assessed by measuring the growth of *P*. *aeruginosa* in TSB with gentamicin. Gentamicin is an effective aminoglycoside antibiotic commonly used in *P*. *aeruginosa* treatments by inhibiting the protein synthesis of the bacteria. The initial density of *P*. *aeruginosa* was 10^5^ CFU/mL according to the guideline of Clinical and Laboratory Standards Institute. The density ratio of the bacteria and the antibody-conjugated particles was maintained at 1:1. After 1 h of incubation, the bacteria were respectively mixed with 0, 0.02, 0.5, and 2 μg/mL gentamicin in the TSB medium at 37°C and 800 rpm for 2 h. A small liquid droplet containing bacterium-binding particles (~0.5 μL, approximately 10^5^ CFU/mL) were taken from a pipette and particles images were recorded every 20 min with a 20× objective to monitor the bacterial activity.

### Statistical analysis

Data from three independent experiments or more are presented as mean ± SEM. One-way ANOVA was performed to determine whether there was a significant difference among groups and a P value of less than 0.05 was considered to be statistically significant.

## Results and Discussions

### Effectiveness and Specificity of the Bead-based Immunoassay

**Fig 3:**
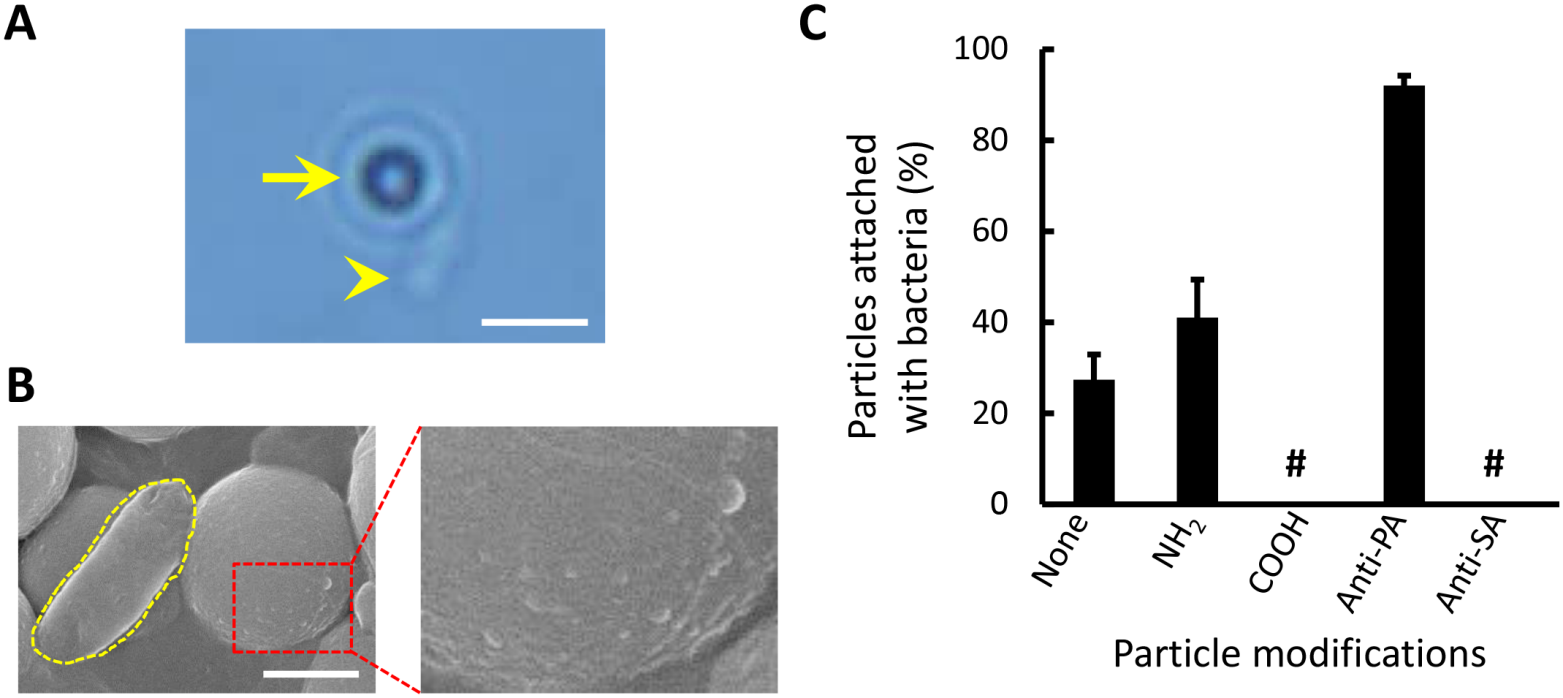
Bacterium-particle bindings. (A) Optical microscopic image of a single *P*. *aeruginosa* bacterium (arrow head) attaching to a 2-μm antibody-conjugated particle (arrow). The scale bar is 5 μm. (B) SEM images of a single *P*. *aeruginosa* bacterium attaching to a 2-μm antibody-conjugated particle and the close-up of the coarse surface of the functionalized particle. The scale bar is 1 μm. (C) Efficacy and specificity of particles with different surface modifications attached to *P*. *aeruginosa*. In the annotation, none (control) means non-modified, NH_2_ means amine-modified, COOH means carboxylate-modified, Anti-PA means anti-*P*. *aeruginosa* polyclonal antibody, Anti-SA means anti-*S*. *aureus* polyclonal antibody, and # means no observation.

An assessment of binding specificity showed that 92 ± 2.2% of the anti-*P*. *aeruginosa* polyclonal antibody modified particles remained attached firmly to *P*. *aeruginosa* after 1 h (Fig 3 and S1 Video). The bacterium-particle complex was verified using a SEM. The rough surfaces of the antibody-conjugated particles were attributed to the matrix formed by the antibodies [28]. The SEM images provided visual evidence showing the successful binding between the bacteria and the particles. The incubation time and binding efficacy here were consistent with those in previous studies based on the bead-based immunoassay [14, 21, 29].

For nonspecific binding, non-modified, carboxylate-modified, amine-modified, and anti-*S*. *aureus* polyclonal antibody modified particles were investigated (Fig 3C). Non-modified particles were treated as a control group in this experiment. The anti-*S*. *aureus* polyclonal antibody modified particles appeared to be clear of nonspecific binding. Conversely, the rest of the modified particles, except the carboxylate-modified particles, showed low degrees of nonspecific binding with *P*. *aeruginosa*. This result proved that particular bacteria can be selectively trapped on particles through antigen-antibody interaction. However, nonspecific binding may remain inevitable if particle surfaces are not treated with a special coating [30].

### Diffusivity Changes of Live and Dead Motile Bacteria

**Fig 4:**
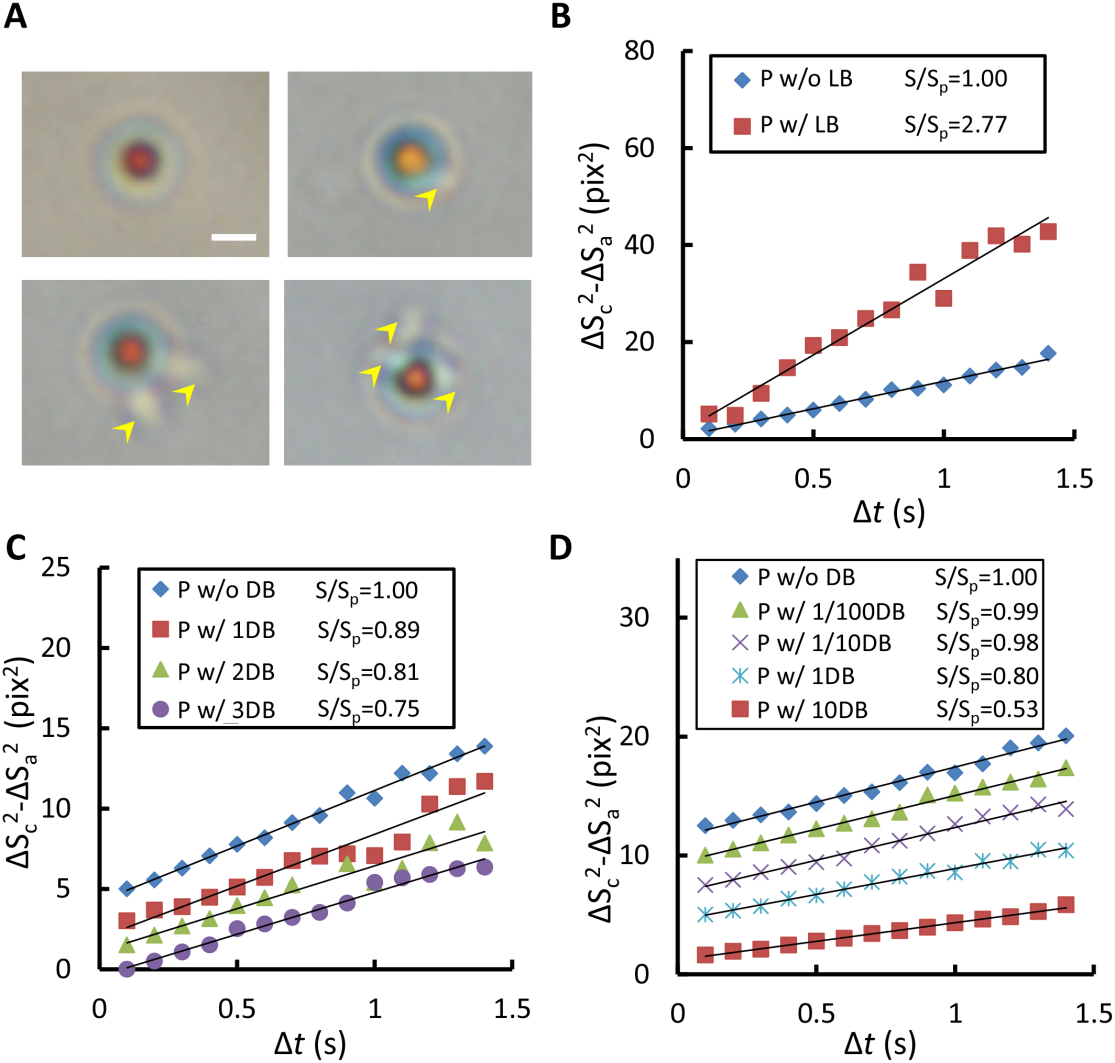
Influences of *P*. *aeruginosa* on Brownian motion of particles. (A) A series of images showing the conjugation of functionalized particles and different numbers of *P*. *aeruginosa* (yellow arrow head). The scale bar is 2 μm. (B) Mean diffusivity values of single particles attached with or without live *P*. *aeruginosa*. (C) Diffusivity values of single particles attached with different numbers of dead *P*. *aeruginosa*. (D) Diffusivity values of populations of particles decline with the increased bacterium density. P means particles, LB means live bacteria, DB means dead bacteria, number before DB means the number of bacteria or the ratio of bacteria to particles, S is the slope of regression line of particles with bacteria, and S_p_ is the slope of regression line of particles without bacteria.

The prior studies have stated that active micro-swimmers may impact particle motion [31–33]. To determine the effect of motile bacteria acting on the particle movement, single particles attached to one, two, three, and four *P*. *aeruginosa* bacteria were respectively analyzed (Fig 4A). Notably, using live and dead bacteria led to different particle behaviors. The free particles in the medium with live bacteria moved more vigorously than did those in the medium with dead bacteria. For the particles attached to live bacteria, no relationship between the diffusivity and the number of bacteria was apparent (S2 Fig and S2 Video). No uniform flow fields were observed because particles bound with live bacteria usually moved in various directions in each image set. Moreover, the combination of different orientations and numbers of bacteria attached to particles makes the trajectory of particles more complex as well. In the measurement, circling, rolling, and spiraling were all observed. Nevertheless, all measurements showed higher diffusivity than those of the free particles (Fig 4B) [22]. A similar phenomenon has also been reported in prior studies, proving that the existence of self-propelled microorganisms, such as bacteria and algae, in a medium can enhance Brownian motion [31–33]. By contrast, the diffusivity of particles decreased monotonically according to the number of dead bacteria they were attached to. From the experimental observations, a higher number of bacteria always resulted in lower diffusivity as compared with one bacterium on single particles because of the larger equivalent particle diameter (Fig 4C and S3 Video; R^2^ ranges from 0.94 to 0.99). Subsequently, the present study measured different bacterium densities to verify the theoretical model. The UV-sterilized *P*. *aeruginosa* was incubated with the antibody-conjugated particles at ratios of 10:1, 1:1, 0.1:1, 0.01:1, and 0:1. The mean diffusivity declined with the increased bacterium density (Fig 4D and S4 Video; R^2^ all over 0.98), which was in favorable agreement with the theoretical prediction.

**Fig 5:**
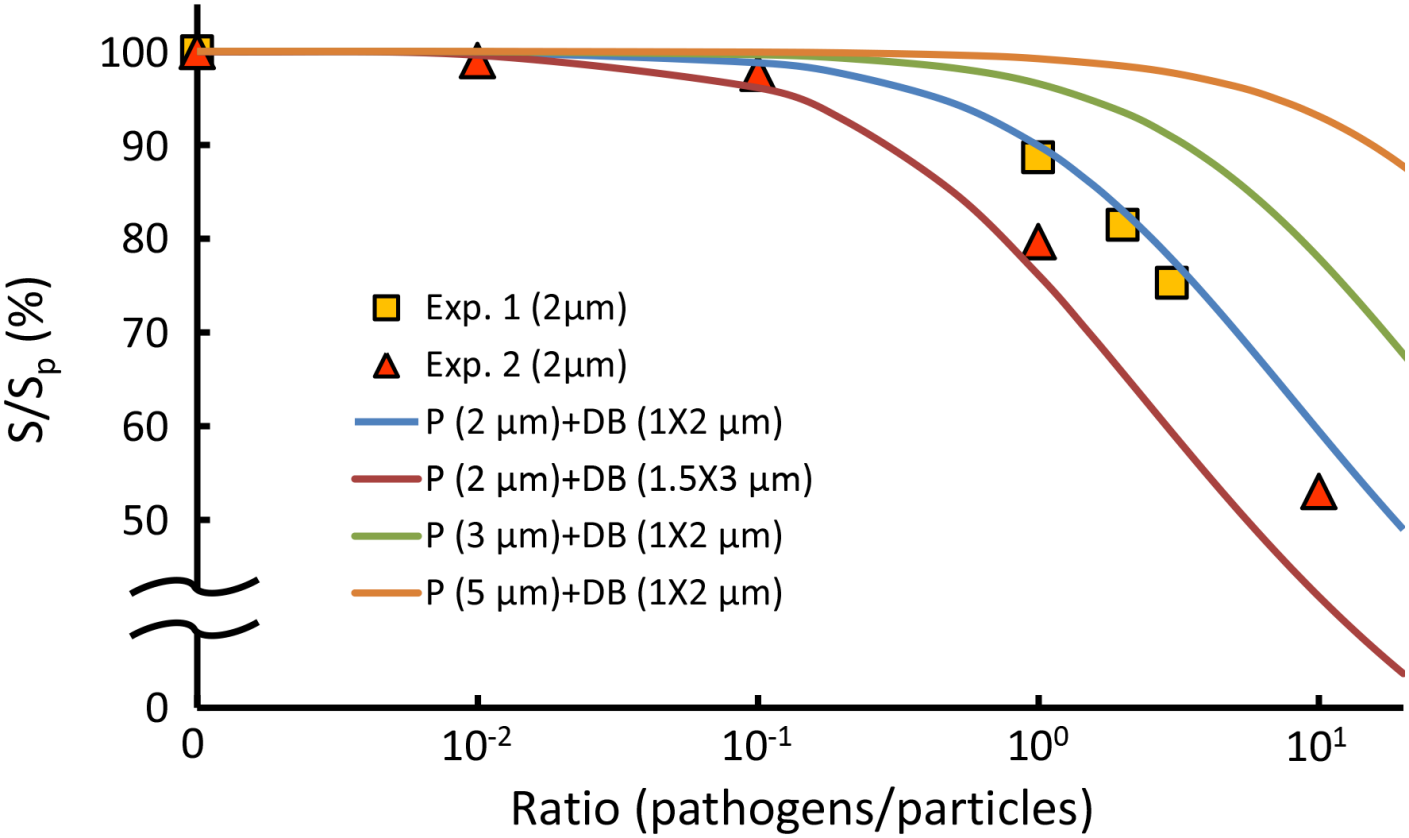
Simulation of the variation in the Brownian motion with different particle sizes and bacterium densities. The symbol P means particles, DB means dead bacteria, S is the slope of regression line of particles with bacteria, and S_p_ is the slope of regression line of particles without bacteria. Exp. 1. denotes the data from Fig 4C. Exp. 2. denotes the data from Fig 4D.

In addition, the experimental data (Fig 5, square and triangle) were compared with those from predicting different particle sizes with respect to different bacterium densities according to equivalent diameter, Eq (7) (Fig 5, solid lines). Overall, the experimental data showed favorable agreements with the predicted curves of 2-μm particles (Fig 5, the blue and red solid lines). Notably, non-spherical particles, such as ellipsoidal and peanut-like colloids, may exhibit biased diffusion due to the coupling effect of translation and rotation. [34–36]. Consequently, the non-spherical bacterium-particle complex are likely to have a slight deviation from the predicted diffusive motion. The deviation can be attributed to the size differences of bacteria and the orientations of the bacteria attached to the particles [14, 34–36]. However, the cross-correlation algorithm is an ensemble average and only the width of the correlation peak is measured. Therefore, the biased diffusion due to the non-spherical shape is negligible. To further mitigate the biased diffusion, we also summed up the correlation peaks by progressively rotating each of them by 90°. Since we only care the relative changes instead of absolute values, the trend will not be altered by the difference in shape. At last, the result showed that the bias and uncertainty were effectively reduced. Accordingly, the theoretical model provides valuable prediction information on the sensitivity and limit of detection of our method when the diameter of the carrier particles or the type of bacteria varies. In general, small particle size indicates high sensitivity but may limit the dynamic range of detection. In contrast to previous estimations of Brownian motion from single particles [14, 19, 20], our method features a rapid measurement of a population of particles, thus yielding an averaged result of the overall microorganism activity. In clinical applications, our method can accurately reflect the response of microorganisms to antibiotics without being negatively influenced by a small fraction of data.

### Rapid Determination of Antimicrobial Susceptibility

**Fig 6:**
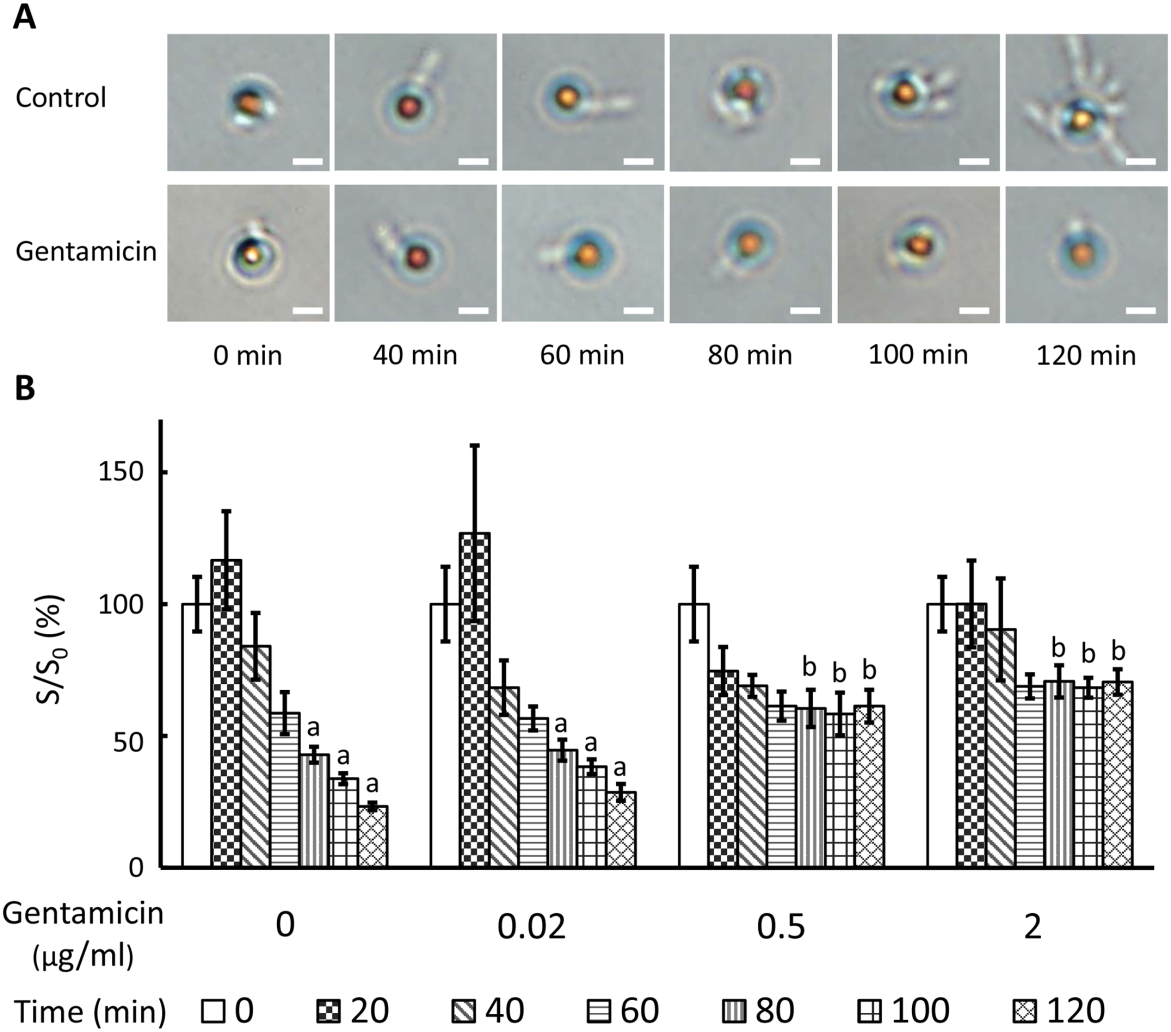
The AST evaluation of *P*. *aeruginosa* in response to gentamicin. (A) Microscopic images of bacteria-binding particles during bacterial growth with or without gentamicin (2 μg/mL). The scale bar is 2 μm. (B) Temporal diffusivity changes of populations of particles attached with bacteria in the presence or absence of gentamicin (0.02, 0.5, and 2 μg/mL). S is the slope of regression line and S_0_ is the regression line slope of particles measured at 0 min. Values at the same time point not sharing a common letter are significantly different (P < 0.05).

On the basis of the experimental results, the technique were then practiced an AST process. Without gentamicin (Control) or with an ineffective concentration of gentamicin (0.02 μg/mL), the *P*. *aeruginosa* bacteria continuously reproduced and attached to the antibody-conjugated particles. In the presence of 0.5 and 2 μg/mL gentamicin, however, the growth of the *P*. *aeruginosa* bacteria was effectively suppressed (Fig 6A). The diffusivity changes of the particles attached to *P*. *aeruginosa* in the control group and in the group of 0.02 μg/mL gentamicin all exhibit increases in the first 20 min, followed by constant decreases to 23.3 and 28.6% of their initial diffusivity values, respectively. By contrast, the diffusivity changes of the particles in the presence of 0.5 and 2 μg/mL gentamicin exhibit slight decreases to 61.3 and 68.8% of their initial diffusivity values, respectively, in the first 60 min and then show no change afterward (Fig 6B). There are significant differences between groups at 80, 100, and 120 min according to the ANOVA analysis. Moreover, at 120 min, the particle diffusivity in the group of 2 μg/mL gentamicin is three times higher than that in the control group.

In other Rapid AST techniques, direct quantification factors including counting by image analysis [8, 17], fluorescence intensity [9], and volume corresponding to the bacteria proliferation [14]; and indirect factors including morphology [8, 10], medium viscosity [15], bacteria disrupted secretion [12], and bacteria metabolism [13] have been utilized to determine the antibiotic susceptibility profiles of bacteria more efficiently. By contrast, our diffusometric platform measures the growth of bacteria using Brownian motion which is subject to the equivalent diameter change resulting from the bacterium binding. As mentioned previously, our method is sensitive to the equivalent diameter change as low as one bacterium (Fig 4C). For a population of particles, the result is an average of the overall microorganism activity, not single ones (Fig 4D). The variations between single particles will be averaged out in the calculation. Therefore, the current method for AST is not only convenient and simple but also sensitive and reliable.

Compared with live *P*. *aeruginosa*, which intensifies Brownian motion, the slightly decreased diffusivity of the particles attached to bacteria in 0.5 and 2 μg/mL gentamicin media was attributed to the bactericidal effect of gentamicin. The variations of diffusivity in this study were subject to several factors including particle sizes [19–21], particle shapes [34–36], bacterial motility [31–33], and viscosity of the medium [15]. In general, Brownian motion is inversely proportional to the equivalent particle diameter if the bacteria are dead. Conversely, live bacteria increase the viscosity of a medium by proliferation and secretion [15, 37, 38], and escalate Brownian motion through frequent collision with particles [31–33]. Before the proliferation reaches a threshold, the Brownian motion of particles is dominated by motile bacteria. When the reproduced bacteria in the medium exceed the threshold, the enlarged particle diameter and increased viscosity then dominate the particle movement. Therefore, the diffusivity here is merely an indicator of the interactions between the mentioned factors that reflects the response of bacteria to antibiotics. To obtain the threshold, a further study will be needed. Thus, according to the results, we define that bacteria are susceptible to an antibiotic at a certain concentration when the diffusivity change of the particles shows a “no decreasing” trend; otherwise, the bacteria are resistant to an antibiotic at a certain concentration.

## Conclusion

In summary, our findings suggest that the diffusivity of particles proportionally declines with the enlarged equivalent particle diameter because of the binding bacteria. The diffusivity of bacterium-particle complexes can be a sensitive indicator of the quantity of particular microorganisms. By analyzing the temporal diffusivity change of particles attached to bacteria, an AST assessment of the response of *P*. *aeruginosa* to gentamicin can be rapidly determined within 2 h. Our study presents a novel technique features a low sample volume (~0.5 μL), a low initial bacteria count (50 CFU per droplet ~ 10^5^ CFU/mL), high sensitivity (one bacterium on single particles), simple fabrication, and rapid AST (within 2 h). Taking advantage of the bead-based immunoassays, multiple types of bacteria can be measured simultaneously by suspending corresponding antibody-modified particles in the medium [21]. In addition, AST evaluations for other bacterial strains can be conducted similarly. The proposed technique will provide insight into achieving rapid and sensitive AST in the near future.

## Supporting Information

S1 Video Bacterium-particle binding. *P*. *aeruginosa* (10^9^ CFU/mL) incubated with antibody-conjugated particles (0.025%, 5.7×10^7^ beads/mL) for 1 h was observed under an optical microscope. In the video, *P*. *aeruginosa* moves with the particles and remains trapped throughout the measurement.
(MPG)

S2 Video Each single particle attaches to different numbers of live bacteria. The video was recorded at least 20 s with a 40× objective.
(AVI)

S3 Video Each single particle attaches to varied numbers of dead bacteria. The video was recorded at least 20 s with a 40× objective.
(AVI)

S4 Video A population of particles with varied ratios of dead bacteria to particles. The video of a population of particles with varied ratios of dead bacteria to particles was recorded at least 20 s with a 20× objective.
(AVI)

S5 Video Rapid bead-based AST. A population of particles attach to live bacteria at ratio 1:1 and then incubate in TSB with 0 and 2 μg/mL gentamicin at 37 ℃ and 800 rpm for 2 hours. The video was recorded at least 20 s with a 20× objective.
(AVI)

S1 Fig Schematic of the particle functionalization. Anti-*P*. *aeruginosa* polyclonal antibody was incubated with EDC and NHS for 15 min. Amine-modified polystyrene beads were washed with MES buffer (pH 5.5) to prevent the aggregation. The polystyrene beads were then functionalized with EDC-NHS activated antibody at 4 ℃ and 800 rpm for 4 h.
(TIF)

S2 Fig Diffusivity of Particles Attached with Live Bacteria. Single particles attached with one, two, three, and four *P*. *aeruginosa* bacteria were respectively analyzed to understand the effect of live bacteria acting on the particle movement. Images were recorded at a time interval (Δ*t*) of 0.1 s with a 40× objective (image sets n = 10 in each group). The diffusivity values of particles attached with live bacteria with Δ*t* were then plotted. To prevent the background variations due to different conditions, all measurements were divided by the diffusivity of the free particles (S_p_). For the particles bound with live bacteria, no apparent relationship between the diffusivity and the number of bacteria. The varied diffusivity values of the particles are likely resulted from the different propulsive forces between bacteria and the orientation of bacteria attached to the particles. Moreover, the combination of different orientations and numbers of bacteria attached to particles complicates the trajectory of particles. Rather than random motion, sometimes circling, rolling, and spiraling were also observed.
(TIF)
