# Supplementary figures and images for: Rapid Bead-Based Antimicrobial Susceptibility Testing by Optical Diffusometry
Source: PLoS One. 2016 Feb 10;11(2):e0148864. doi: 10.1371/journal.pone.0148864 (PMC4749332; doi:10.1371/journal.pone.0148864)

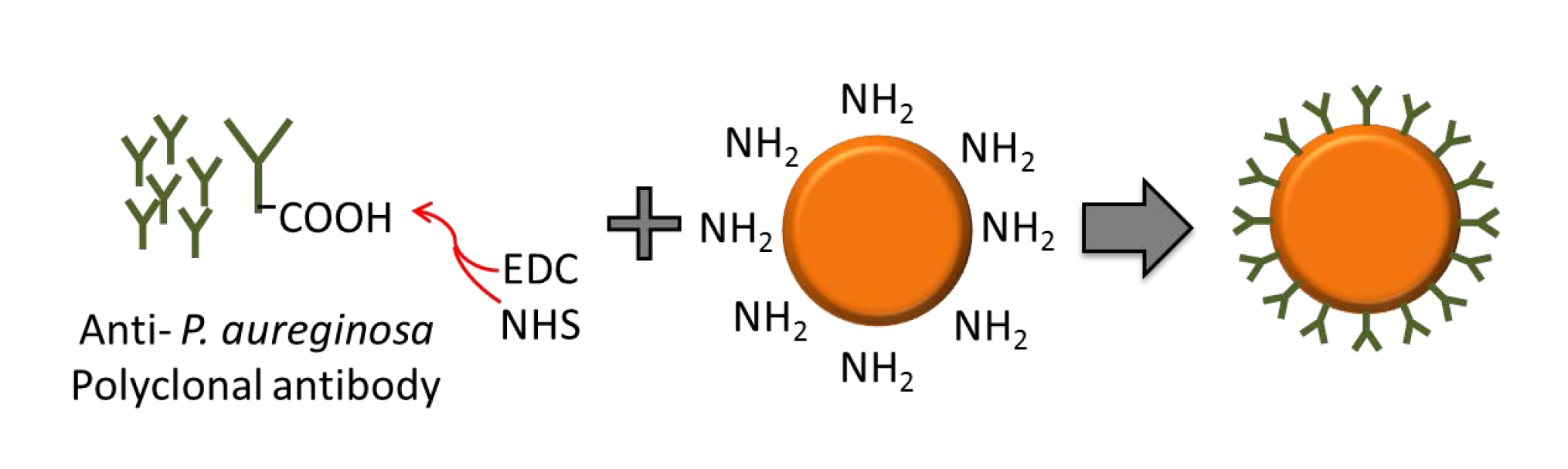

Supplement: S1 Fig — Anti-P. aeruginosa polyclonal antibody was incubated with EDC and NHS for 15 min. Amine-modified polystyrene beads were washed with MES buffer (pH 5.5) to prevent the aggregation. The polystyrene beads were then functionalized with EDC-NHS activated antibody at 4 ℃ and 800 rpm for 4 h. (TIF) [file pone.0148864.s006.tif]

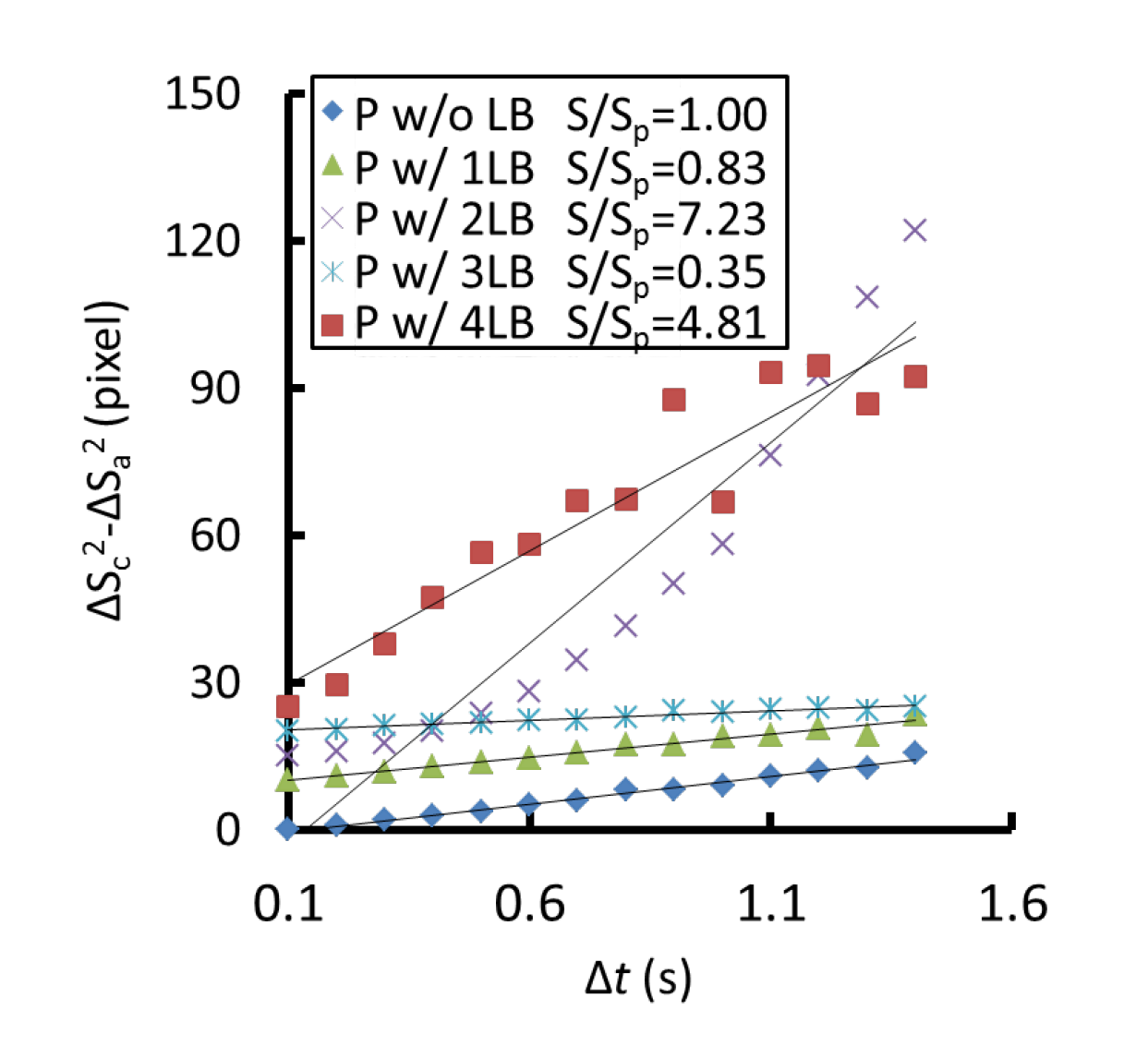

Supplement: S2 Fig — Single particles attached with one, two, three, and four P. aeruginosa bacteria were respectively analyzed to understand the effect of live bacteria acting on the particle movement. Images were recorded at a time interval (Δt) of 0.1 s with a 40× objective (image sets n = 10 in each group). The diffusivity values of particles attached with live bacteria with Δt were then plotted. To prevent the background variations due to different conditions, all measurements were divided by the diffusivity of the free particles (Sp). For the particles bound with live bacteria, no apparent relationship between the diffusivity and the number of bacteria. The varied diffusivity values of the particles are likely resulted from the different propulsive forces between bacteria and the orientation of bacteria attached to the particles. Moreover, the combination of different orientations and numbers of bacteria attached to particles complicates the trajectory of particles. Rather than random motion, sometimes circling, rolling, and spiraling were also observed. (TIF) [file pone.0148864.s007.tif]
